# Formulation and In Vitro Evaluation of Red Palm Oil with *Rhinacanthus nasutus*, *Curcuma longa*, *Zingiber montanum*, and *Zingiber officinale* Extracts as a Possible Pet Shampoo Formula with Antibacterial, Antifungal, and Anti-Inflammatory Activities

**DOI:** 10.3390/vetsci13070712

**Published:** 2026-07-20

**Authors:** Natthrit Roekngam, Sunisa Khongthong, Ricadonna Raissa, Bongkoch Chonglomkrod, Sara Niae

**Affiliations:** 1 Faculty of Veterinary Science, Rajamangala University of Technology Srivijaya, Nakhon Si Thammarat 80240, Thailand; natthrit.r@rmutsv.ac.th (N.R.); sunisa.k@rmutsv.ac.th (S.K.); bongkoch.c@rmutsv.ac.th (B.C.); 2 Faculty of Veterinary Medicine, Universitas Brawijaya, Malang 65151, East Java, Indonesia; ricadonnaraissa@ub.ac.id

**Keywords:** red palm oil, *Rhinacanthus nasutus*, *Curcuma longa*, *Zingiber montanum*, *Zingiber officinale*, antimicrobial activity, COX-2 inhibition, veterinary dermatology

## Abstract

Bacterial and fungal skin infections are common in companion animals and often require antimicrobial therapy, contributing to antimicrobial resistance. This study developed shampoos containing red palm oil (RPO) and extracts of *Rhinacanthus nasutus* (RN), *Curcuma longa* (CL), *Zingiber montanum* (ZM), and *Zingiber officinale* (ZO), which provide antibacterial, antifungal, antioxidant, and anti-inflammatory activities. Extracts were obtained using MAE and were evaluated for phytochemical composition, total phenolic content, antioxidant activity (DPPH assay), anti-inflammatory activity (COX-2 inhibition), and antimicrobial activity. RPO contained high levels of vitamin E and β-carotene. Rhinacanthin, curcumin, terpinen-4-ol, and 6-gingerol were the major phytochemical constituents of RN, CL, ZM, and ZO extracts, respectively. All extracts significantly reduced COX-2 expression (*p* < 0.05), while RN exhibited the strongest antioxidant activity (DPPH IC_50_ = 15.23 ± 0.53 µg/mL). RN and ZM demonstrated the strongest antibacterial activity against *Staphylococcus aureus* and methicillin-resistant *Staphylococcus aureus* (MIC = 8 µg/mL; MBC = 8–16 µg/mL), while ZO exhibited the strongest antifungal activity against *Malassezia pachydermatis* and *Microsporum gypseum* (MIC = 2, 2 µg/mL; MFC = 2, 16 µg/mL). The developed shampoos containing RPO and the four herb extracts showed suitable pH values, stable viscosity, antibacterial inhibition, and antifungal inhibition (*p* < 0.05).

## Simple Summary

Antimicrobial resistance associated with prolonged antibiotic use has become a growing concern in companion animal medicine. This study investigated the potential of red palm oil and four Thai medicinal herbs as natural ingredients for dog and cat shampoos. The herbal extracts were evaluated for their antioxidant, antibacterial, antifungal, and anti-inflammatory activities. All extracts exhibited antioxidant activity and demonstrated antibacterial, antifungal, and anti-inflammatory effects, and antimicrobial activity was maintained after they were incorporated into the shampoo formulations. The developed shampoos also showed pH and viscosity values suitable for topical application. These findings highlight the potential of RPO and Thai herbal extracts as natural, multifunctional ingredients for veterinary dermatological products. The use of these natural ingredients may help decrease the long-term use of antibiotics and reduce the development of antimicrobial resistance.

## 1. Introduction

Recently, the number of pets, particularly dogs and cats, has steadily increased [1] because of their positive effects on human mental health [2], and pets are regarded as family members [3,4,5]. However, pets are often associated with skin diseases [6,7,8] caused by bacteria, fungi, and yeasts: for example, *Staphylococcus* spp., *Microsporum* spp., and *Malassezia* spp. [9,10]. These microorganisms can be transmitted from animals to humans and may also spread in the environment [11,12,13,14]. To treat these skin diseases, veterinarians commonly use antibiotics and antifungal drugs as systemic or topical antimicrobial agents in shampoo for the long term [15,16]. However, the use of these medications may lead to drug toxicity and antimicrobial resistance (AMR) [17,18], which have become a major global concern in both human and veterinary medicine [19]. Therefore, alternative approaches for the management of skin diseases in companion animals are needed. Natural products that are safe and effective may help reduce the use of conventional antibiotics and antifungal drugs [20,21,22]. Although herbal shampoos are commercially available, most formulations are primarily intended for ectoparasite control rather than for the management of microbial skin infections [23,24]. A shampoo formulated with red palm oil (RPO) and Thai herbal extracts may be a promising alternative to veterinary skin-care products. Red palm oil, the main ingredient of the shampoo, contains high levels of β-carotene, vitamin A, and vitamin E, which have antioxidant and skin-protective properties [25,26,27,28]. In addition, RPO has antimicrobial activity against Gram-positive bacteria, which are important causes of skin diseases in pets [25]. Furthermore, the palm oil industry produces byproducts such as kernel cake and palm shell, which are currently underutilized [29]. The use of palm oil-derived products in veterinary applications may help reduce waste and promote sustainable production. Therefore, the development of pet shampoo containing RPO and Thai herbal extracts may provide a natural, safe, and sustainable option for veterinary dermatological care.

*Rhinacanthus nasutus* (RN; Thong phan chang), *Curcuma longa* (CL; Turmeric), *Zingiber montanum* (ZM; Cassumunar ginger), and *Zingiber officinale* (ZO; Ginger) are Thai medicinal plants that contain bioactive compounds with established antimicrobial, antioxidant, and anti-inflammatory activities [30,31,32,33]. Previous investigations have largely focused on individual herbal extracts, primarily in human medicine or as oral supplements in animals [34,35,36,37]. To our knowledge, no study has reported the incorporation of the best of these four Thai herbal extracts in combination with RPO into a shampoo formulation intended for companion animal skin care. The incorporation of Thai herbal extracts into shampoo formulations may improve the effectiveness of the shampoo for the treatment of skin diseases in companion animals. In addition, it may help reduce the use of antibiotics and antifungal drugs, thereby reducing the risk of drug-related side effects.

Microwave-assisted extraction (MAE) is a suitable technique for extracting bioactive compounds from medicinal plants and lipid-based materials [38,39]. Compared with conventional extraction methods, such as maceration and Soxhlet extraction, the MAE method provides high extraction yields, requires less solvent, and helps protect heat-sensitive compounds [40,41,42]. In addition, MAE is an environmentally friendly extraction technique with applications in pharmaceutical, cosmetic, and veterinary product development [43,44,45,46,47].

Therefore, this study hypothesized that the combination of RPO and selected Thai herbal extracts would provide antimicrobial, antifungal, antioxidant, and anti-inflammatory activities suitable for topical application in companion animals to develop plant-based, environmentally friendly, and sustainable alternatives for veterinary dermatological care. This study aimed to (1) extract bioactive compounds from RPO and selected Thai medicinal plants using MAE; (2) characterize the chemical and biological properties of the extracts and determine their appropriate concentrations for shampoo formulation; and (3) develop and evaluate an RPO-based herbal shampoo with potential applications for companion animal skin care.

## 2. Materials and Methods

### 2.1. Ethical Considerations

All experiments were conducted entirely in vitro in accordance with institutional biosafety regulations and standard laboratory practice guidelines and did not involve any live animal subjects or clinical samples. The microbial strains used in this study were established and previously identified laboratory isolates. Therefore, approval from an animal ethics and biosafety committee was not required for this study. During manuscript preparation, ChatGPT-5.5 Instant (OpenAI) was used solely to assist with English grammar editing. The AI tool was not used for study design, data analysis, interpretation of results, or the generation of scientific content. All content was reviewed, verified, and approved by the authors.

### 2.2. Study Period and Location

Oil palm mesocarp residues used for red palm oil (RPO) extraction were obtained from the Tha Sa Ba Oil Palm Enterprise Group, Trang Province, Thailand. Thai medicinal plants, including *Rhinacanthus nasutus* (leaves), *Curcuma longa* (rhizomes), *Zingiber montanum* (rhizomes), and *Zingiber officinale* (rhizomes), were collected from cultivated sources in southern Thailand between August and September 2024. All plant materials were authenticated according to the *Thai Herbal Pharmacopoeia* by a qualified plant taxonomist, and voucher specimens were deposited in the Herbarium of the Faculty of Veterinary Science, Rajamangala University of Technology Srivijaya, under accession numbers RN-VET-001, CL-VET-002, ZM-VET-003, and ZO-VET-004, respectively.

This study was conducted from August 2024 to May 2026. All laboratory work, including extraction, phytochemical characterization, antioxidant and anti-inflammatory assays, antimicrobial susceptibility testing, shampoo formulation, and physicochemical stability evaluation, was carried out at the Faculty of Veterinary Science, Rajamangala University of Technology Srivijaya, Thung Yai District, Nakhon Si Thammarat 80240, Thailand (8.306° N, 99.731° E), under controlled laboratory conditions.

### 2.3. Plant Authentication

Fresh RN, CL, ZM, and ZO plant materials were collected from cultivated sources in southern Thailand between August and September 2024. Botanical identification followed the *Thai Herbal Pharmacopoeia* and was performed by a qualified plant taxonomist at the Faculty of Veterinary Science, Rajamangala University of Technology Srivijaya.

Voucher specimens were deposited in the Herbarium of the same faculty under accession numbers RN-VET-001 (*R. nasutus*), CL-VET-002 (*C. longa*), ZM-VET-003 (*Z. montanum*), and ZO-VET-004 (*Z. officinale*) and retained for future reference and verification. Only healthy, disease-free plant materials without visible mechanical damage or fungal contamination were selected for extraction to ensure sample quality and experimental consistency.

### 2.4. Preparation of RPO from Palm Fruit Residue

Red palm oil (RPO) was extracted from dried oil palm mesocarp residue via microwave-assisted extraction (MAE). Dried mesocarp residue (100 g) was mixed with 100 mL of absolute ethanol (1:1, *w*/*v*) in a microwave-resistant glass container and extracted in a domestic microwave oven (MS23K3513AW/ST, Samsung Electronics Co., Ltd., Suwon, Republic of Korea) at 360 W for three 5 min cycles [48], with cooling intervals between cycles to prevent overheating. The mixture was filtered through Whatman No. 1 filter paper, and the filtrate was concentrated under reduced pressure using a rotary evaporator (Rotavapor R-100, BÜCHI Labortechnik AG, Flawil, Switzerland) at 40 °C to remove residual solvents. The resulting RPO was weighed and stored in amber glass bottles at 4 °C until analysis. Extractions were performed in triplicate (*n* = 3 independent extractions), and yield was calculated as follows:

Yield (%) = (Weight of dried extract/Weight of dried raw material) × 100

### 2.5. Preparation of Herbal Extraction

Authenticated leaves of RN and rhizomes of CL, ZM, and ZO were thoroughly washed with running tap water followed by distilled water to remove adhering soil and foreign materials. Plant materials were cut into small pieces (approximately 0.5–1.0 cm), oven-dried at 60 °C for 48 h until constant weight, and ground into fine powder using a laboratory grinder. The resulting powders were passed through a 40-mesh sieve to obtain a uniform particle size and stored in airtight light-resistant containers at 4 °C until extraction. For each extraction, 100 g of dried plant powder was mixed with 1000 mL of absolute ethanol (solid-to-solvent ratio 1:10, *w*/*v*) in a microwave-resistant glass vessel. Extraction was performed using microwave-assisted extraction (MAE) in a domestic microwave oven (MS23K3513AW/ST, Samsung Electronics Co., Ltd., Suwon, Republic of Korea) at 360 W for three 5 min extraction cycles, with approximately 5 min cooling intervals between each cycle to minimize thermal degradation of heat-sensitive phytochemicals. After extraction, the mixtures were filtered through Whatman No. 1 filter paper, and the filtrates were concentrated under reduced pressure using a rotary evaporator (Rotavapor R-100, BÜCHI Labortechnik AG, Flawil, Switzerland) at 40 °C until complete solvent removal. Crude extracts were weighed to determine extraction yield and subsequently transferred into amber glass bottles, flushed with nitrogen when appropriate, sealed tightly, and stored at 4 °C until further phytochemical and biological analyses. All extractions were performed independently in triplicate (*n* = 3). The extraction yield was calculated using the following equation:

Yield (%) = (Weight of dried extract/Weight of dried plant material) × 100

Different solid-to-solvent ratios were employed for RPO and herbal extractions because RPO extraction targeted lipid-rich oil palm mesocarp residue, whereas herbal extraction was designed to recover predominantly polar phytochemical constituents from dried medicinal plant materials.

### 2.6. Vitamin A Analysis

Vitamin A content in RPO was determined spectrophotometrically using a method modified from Subramanyam and Parrish [49]. A 0.5 g sample was saponified with 5 mL of 10% (*w*/*v*) KOH at 65 °C for 30 min. After cooling, the mixture was extracted with hexane (2:1, hexane:sample solution) and vortex-mixed. The organic phase was collected and evaporated to dryness, and the residue was reconstituted in 2 mL of dichloromethane (DCM) and mixed with 2 mL of trichloroacetic acid (TCA) reagent. Absorbance was measured at 620 nm within 2 min using a UV–Vis spectrophotometer.

A calibration curve was prepared with vitamin A standards over 0–10 ppm and showed good linearity (R^2^ > 0.999). The limit of detection (LOD) and limit of quantification (LOQ) were 0.02 and 0.05 ppm, respectively. Vitamin A content was calculated from the calibration curve and expressed as µg/g sample. Analyses were performed in triplicate (*n* = 3), with results reported as mean ± standard deviation (SD).

### 2.7. Vitamin E Analysis

Vitamin E content was determined according to AOAC Official Method 971.30 with minor modifications. A 0.5 g sample was saponified with 5 mL of 10% (*w*/*v*) KOH at 65 °C for 30 min and then extracted with hexane at twice the sample volume. The organic layer was evaporated to dryness and reconstituted in 5 mL of ethanol.

For color development, 1 mL of bathophenanthroline solution, 1 mL of ferric chloride (FeCl_3_) solution, 1 mL of concentrated phosphoric acid, and 2 mL of ethanol were added sequentially. The reaction mixture was protected from light, and absorbance was measured at 534 nm against an ethanol blank.

Quantification used an α-tocopherol calibration curve (0–10 ppm), which showed excellent linearity (R^2^ > 0.999). LOD and LOQ were 0.02 and 0.05 ppm, respectively. Vitamin E content was expressed as µg/g sample. Measurements were performed in triplicate (*n* = 3), with data reported as mean ± SD.

### 2.8. Fatty Acid Composition Analysis

The fatty acid composition of RPO was analyzed via gas chromatography–mass spectrometry (GC–MS) (Agilent 7890B GC coupled with a 5977B Mass Selective Detector, Agilent Technologies, Santa Clara, CA, USA). Crude lipids were converted to fatty acid methyl esters (FAMEs) via acid-catalyzed transesterification using 2% methanolic sulfuric acid at 60 °C for 60 min, following standard procedures [50,51]. After cooling, FAMEs were extracted with hexane, washed with saturated sodium chloride solution, and evaporated under a gentle nitrogen stream.

Separation was performed on a DB-5MS capillary column (30 m × 0.25 mm i.d. × 0.25 µm film thickness; Agilent Technologies, Santa Clara, CA, USA) with helium as a carrier gas at a constant flow of 1.0 mL/min. The injector temperature was 250 °C with a 1:10 split ratio. The oven program began at 60 °C (held 2 min), increased to 200 °C at 10 °C/min, and then increased to 250 °C, where it was held for 10 min to ensure complete elution of long-chain fatty acids. Mass spectrometric detection used electron impact (EI) ionization at 70 eV, with the ion source at 230 °C and the transfer line at 280 °C. Data were acquired in full-scan mode (*m*/*z* 40–450).

Fatty acids were identified by comparing retention times and mass spectra with certified FAME standards and the NIST mass spectral library [52]. Quantification was based on relative peak area normalization and expressed as a percentage (%) of total fatty acids. Analyses were performed in triplicate (*n* = 3), with results reported as mean ± SD.

### 2.9. Determination of Total Phenolic Content

The total phenolic content (TPC) of herbal extracts was determined using the Folin–Ciocalteu colorimetric method, which quantifies the reducing capacity of phenolic compounds through electron-transfer reactions [49], and it is well suited to evaluating phenolic constituents in plant-based veterinary formulations. This method was applied consistently across all extracts. Briefly, 0.1 mL of each extract was diluted with 0.4 mL of ethanol, followed by the addition of a 2 mL sodium carbonate solution (75 g/L) to create an alkaline environment for the reduction of the Folin–Ciocalteu reagent. After mixing, 0.1 mL of Folin–Ciocalteu reagent (2.0 M) was added to initiate chromophore formation. The mixture was incubated at room temperature in the dark for 30 min to prevent light-induced degradation, and absorbance was measured at 750 nm.

A calibration curve was constructed using gallic acid standards (0–200 mg/L), and TPC was expressed as milligrams of gallic acid equivalents per gram of dry extract (mg GAE/g), following established protocols [50]. Samples were analyzed in triplicate, and the results were presented as mean ± SD.

### 2.10. Antioxidant Activity (DPPH Assay)

Antioxidant activity of RPO and herbal extracts was evaluated using the 2,2-diphenyl-1-picrylhydrazyl (DPPH) radical scavenging assay, following previously described methods [53,54]. A 0.2 mM DPPH solution was prepared in ethanol and stored in the dark at 4 °C until use.

For herbal extracts, dried crude extracts were completely dissolved in absolute ethanol to prepare the desired concentrations. Because red palm oil (RPO) is a lipid-rich matrix and is not completely soluble in ethanol, RPO samples were first dispersed in absolute ethanol by vigorous vortex mixing for 1 min, followed by ultrasonic treatment (40 kHz, 5 min) to obtain a homogeneous dispersion immediately before analysis. The dispersed samples were mixed thoroughly prior to each measurement to minimize phase separation during the assay. Therefore, the term “dispersed” rather than “dissolved” is used throughout this manuscript when referring to RPO sample preparation.

For the assay, 0.1 mL of the sample solution was mixed with 3.0 mL of DPPH solution and incubated at room temperature in the dark for 30 min. Absorbance was measured at 517 nm. Ethanol mixed with DPPH solution served as the negative control, and ascorbic acid served as the positive control. DPPH radical scavenging activity was calculated as follows:

% Radical scavenging activity = [(A_0_ − A_1_)/A_0_] × 100

where A_0_ is the absorbance of the control reaction, and A_1_ is the absorbance of the sample reaction.

Measurements were performed in triplicate (*n* = 3) and expressed as mean ± SD. The concentration required to inhibit 50% of DPPH radicals (IC_50_) was determined via nonlinear regression using GraphPad Prism (Version 10.0, GraphPad Software Inc., San Diego, CA, USA). Lower IC_50_ values indicate higher antioxidant activity.

### 2.11. Antibacterial Activity

Antibacterial activities of RPO and herbal extracts were evaluated against five clinically relevant strains: *Staphylococcus aureus* ATCC 25923, methicillin-resistant *S. aureus* (MRSA), *Staphylococcus epidermidis*, *Escherichia coli* ATCC 25922, and *Pseudomonas aeruginosa* ATCC 10145. Strains were cultured on Tryptic Soy Agar (TSA) at 37 °C for 24 h and then transferred to Mueller–Hinton Broth (MHB) for overnight incubation. Bacterial suspensions were adjusted to a 0.5 McFarland standard (approximately 1 × 10^8^ CFU/mL) and diluted to approximately 1 × 10^6^ CFU/mL according to CLSI guidelines [55].

Antibacterial activity was determined by the broth microdilution method following CLSI guideline M07-A10. Serial two-fold dilutions of each extract were prepared in sterile 96-well microplates. Vancomycin served as the positive control for Gram-positive bacteria, and gentamicin served as the positive control for Gram-negative bacteria; 1% (*v*/*v*) DMSO served as the negative control. The final DMSO concentration in all wells did not exceed 1% and showed no inhibitory effect on bacterial growth.

Each well received 100 µL of bacterial suspension and was incubated at 37 °C for 18 h, followed by addition of 20 µL of resazurin solution (20 mg/mL) and a further 5 h incubation. Bacterial growth was assessed by absorbance at 750 nm. The minimum inhibitory concentration (MIC) was defined as the lowest concentration inhibiting bacterial growth by at least 90% relative to the untreated control.

For the minimum bactericidal concentration (MBC), aliquots from wells showing no visible growth were subcultured onto TSA plates and incubated at 37 °C for 24 h; MBC was defined as the lowest concentration yielding no visible colony growth. All experiments were performed in triplicate (*n* = 3).

### 2.12. Antifungal Activity

Antifungal activities of RPO and herbal extracts were evaluated against *Malassezia pachydermatis*, *Microsporum canis*, and *Microsporum gypseum*, which are common fungal pathogens associated with dermatological disease in companion animals [13,18]. Strains were cultured in Brain Heart Infusion (BHI) broth to the logarithmic phase and then adjusted to a 0.5 McFarland standard (approximately 1 × 10^6^ CFU/mL) and diluted to approximately 1 × 10^5^ CFU/mL.

Antifungal activity was evaluated by broth microdilution according to CLSI guideline M38-A2 [56]. Serial two-fold dilutions of each extract were prepared in sterile 96-well microplates. Clotrimazole served as the positive control and 1% (*v*/*v*) DMSO served as the negative control; DMSO concentration did not exceed 1% in any well and had no inhibitory effect on fungal growth.

Each well received 100 µL of fungal suspension and was incubated at 37 °C for 18–24 h, followed by the addition of 20 µL of resazurin solution (20 mg/mL) and a further 5 h incubation. Absorbance was measured at 750 nm. MIC was defined as the lowest concentration inhibiting fungal growth by at least 90% relative to the untreated control.

For the minimum fungicidal concentration (MFC), aliquots from wells showing no visible growth were subcultured onto Sabouraud Dextrose Agar (SDA) plates and incubated at 37 °C for 48 h; MFC was defined as the lowest concentration yielding no visible fungal colony growth. All experiments were performed in triplicate (*n* = 3).

### 2.13. Shampoo Formulation

Based on the antimicrobial, antifungal, antioxidant, and anti-inflammatory screening results, two prototype topical shampoo formulations were developed for companion animals, consisting of one formulation intended for dogs and another for cats. Separate formulations were designed because canine and feline skin differ in several physiological characteristics, including skin surface pH, epidermal barrier function, sebaceous gland activity, grooming behavior, and susceptibility to topical products [57]. Dogs generally tolerate slightly more alkaline topical formulations, whereas cats possess relatively delicate skin and frequently groom themselves after bathing, thereby increasing the possibility of accidental oral exposure to residual shampoo [57]. Therefore, species-specific formulation parameters were considered during product development to improve compatibility with the intended animal while maintaining identical active phytochemical ingredients.

Both formulations contained the same bioactive ingredients, including red palm oil (RPO), *Rhinacanthus nasutus* (RN), *Curcuma longa* (CL), *Zingiber montanum* (ZM), and *Zingiber officinale* (ZO). Selection of these ingredients was based on the biological screening results obtained in the present study. RN and ZM were incorporated as the principal antibacterial components because they exhibited the strongest inhibitory activity against *Staphylococcus aureus*, methicillin-resistant *S. aureus* (MRSA), and *Staphylococcus epidermidis*. ZO was selected as the primary antifungal ingredient owing to its superior activity against *Malassezia pachydermatis* and *Microsporum gypseum*, while CL was included primarily for its potent anti-inflammatory activity through suppression of COX-2 expression. RPO served not only as the lipid-rich carrier but also as a functional ingredient by providing β-carotene, vitamin A, vitamin E, essential fatty acids, and additional antioxidant compounds that may contribute to skin protection and maintenance of skin barrier integrity [25]. The formulation was designed to combine extracts with complementary biological properties rather than to demonstrate pharmacodynamic synergism.

The shampoo base was prepared by dispersing sodium lauryl ether sulfate (SLES) and cocamidopropyl betaine in purified water under continuous mechanical stirring at room temperature. Glycerin was subsequently incorporated as a humectant to improve skin hydration and reduce moisture loss following topical application [58]. Herbal extracts and RPO were separately dispersed prior to gradual incorporation into the shampoo base under continuous stirring until a homogeneous formulation was obtained. Potassium sorbate was then added as a preservative, and citric acid solution was used to adjust the final pH where necessary.

Although both formulations contained identical active ingredients, slight modifications were made to the surfactant composition and final pH to better accommodate species-specific skin physiology. The dog shampoo was adjusted to approximately pH 7.7, whereas the cat shampoo was adjusted to approximately pH 7.0, values selected according to published recommendations regarding canine and feline skin physiology and topical veterinary formulations [57]. These pH values were selected to maximize compatibility with the normal skin environment while minimizing the risk of disrupting epidermal barrier function during repeated topical application [59]. The final volume of each formulation was adjusted with purified water and mixed until completely homogeneous. The detailed composition of each formulation is presented in Table A1.

The formulated shampoos were transferred into opaque high-density polyethylene (HDPE) containers to minimize light-induced degradation of carotenoids and other light-sensitive phytochemicals [25], and they were stored at 25 ± 2 °C until physicochemical characterization, antimicrobial and antifungal evaluation, and preliminary stability assessment.

Based on the antimicrobial screening results, RN and ZM were selected as the principal antibacterial ingredients, whereas ZO was incorporated as the principal antifungal ingredient. The formulation was designed to combine extracts with complementary biological properties rather than to demonstrate pharmacodynamic synergism.

### 2.14. Evaluation of Shampoo Formulations

The shampoo formulations were evaluated for physicochemical stability and biological activity. Stability testing was conducted under real-time (25 ± 2 °C) and accelerated (40 ± 2 °C) storage conditions for 3 months. Appearance, color, odor, pH, viscosity, and phase separation were evaluated at predetermined intervals throughout storage.

Physical stability was additionally assessed by centrifugation: 10 mL of each formulation was centrifuged at 3000 rpm for 30 min at room temperature and then visually inspected for phase separation, precipitation, or creaming. pH was measured using a calibrated digital pH meter, and viscosity was determined using a Brookfield viscometer. Stability was considered acceptable when no significant changes in appearance, pH, viscosity, or phase separation occurred during storage.

Biological activity of the final formulations was re-evaluated after formulation to confirm that antimicrobial efficacy was retained following incorporation into the surfactant matrix. Antibacterial activity was determined by broth microdilution according to CLSI guidelines [55,60], and antifungal activity was evaluated against *M. canis*, *M. gypseum*, and *M. pachydermatis* using the procedures described above. All experiments were performed in triplicate (*n* = 3).

### 2.15. Statistical Analysis

All experiments were performed independently in triplicate (*n* = 3), and the results were expressed as mean ± SD. Statistical analyses were conducted using IBM SPSS Statistics (Version 29.0; IBM Corp., Armonk, NY, USA). Normality of data distribution was assessed using the Shapiro–Wilk test prior to analysis. Differences among treatment groups were evaluated via one-way analysis of variance (ANOVA), with significant differences further compared using Tukey’s multiple-comparison post hoc test. For gene expression analysis, relative mRNA expression levels were calculated using the 2^−ΔΔCt^ method, and statistical comparisons were performed using one-way ANOVA followed by Tukey’s multiple-comparison test in GraphPad Prism version 11.0.2 for macOS (GraphPad Software, Boston, MA, USA). GraphPad Prism was also used for graphical presentation of the gene expression data. Differences were considered significant at *p* < 0.05.

## 3. Results

### 3.1. Extraction Yield and Phytochemical Analysis

Microwave-assisted extraction successfully recovered red palm oil (RPO) and bioactive compounds from the selected Thai medicinal plants. The extraction yield of RPO from oil palm mesocarp residue was 42.02 ± 1.77% (*w*/*w*), giving a characteristic dark-red oil rich in carotenoids and lipid-soluble antioxidants. Extraction yields of the medicinal plants were 27.40 ± 1.77% for *Rhinacanthus nasutus* (RN), 29.10 ± 8.91% for *Curcuma longa* (CL), 34.00 ± 2.83% for *Zingiber montanum* (ZM), and 14.60 ± 0.57% for *Zingiber officinale* (ZO) (mean ± SD, *n* = 3).

High-performance liquid chromatography (HPLC) analysis confirmed the presence of major phytochemical marker compounds in all extracts (Table 1). CL showed the highest concentration of marker compound among the extracts, while RN and ZM contained moderate levels of their respective bioactive constituents.

Spectrophotometric analysis showed that RPO contained 1.88 ± 0.05 ppm vitamin A, 788.94 ± 12.45 ppm β-carotene, and 810.00 ± 15.32 ppm vitamin E, indicating that RPO is a rich source of natural antioxidants with potential application in topical formulations.

The fatty acid composition of mesocarp-derived RPO was comparable to that of commercial crude RPO (Table 2). Palmitic acid was the predominant fatty acid, accounting for 54.36 ± 0.82% of total fatty acids in mesocarp-derived RPO versus 40.17 ± 0.64% in crude RPO. Other major fatty acids included oleic acid (29.74 ± 0.67% and 40.08 ± 0.81%, respectively), linoleic acid (6.60 ± 0.19% and 10.27 ± 0.25%), stearic acid (4.08 ± 0.11% and 4.85 ± 0.13%), linolenic acid (0.17 ± 0.01% and 0.35 ± 0.02%), arachidic acid (0.33 ± 0.02% and 0.38 ± 0.03%), and eicosanoic acid (0.10 ± 0.01% and 0.14 ± 0.01%). Overall, the fatty acid profile of mesocarp-derived RPO closely resembled that of commercial crude RPO, suggesting that the extraction process effectively preserved the lipid composition of the oil.

### 3.2. Bioactive Properties of Herbal Extracts

Bioactive properties of the selected Thai medicinal plant extracts were evaluated through total phenolic content (TPC), antioxidant activity, and anti-inflammatory activity assays.

The TPC and antioxidant activity of Thai medicinal plant extracts are shown in Table 3. TPC values differed significantly among the extracts (*p* < 0.05). CL exhibited the highest TPC (242.00 ± 5.12 mg GAE/g DW), followed by RN (121.94 ± 7.69 mg GAE/g DW), ZM (117.34 ± 4.23 mg GAE/g DW), and ZO (89.45 ± 4.53 mg GAE/g DW). The high phenolic content of CL suggests a substantial contribution of phenolic compounds to its biological activities.

Antioxidant activity, determined by the DPPH radical scavenging assay, revealed strong free radical scavenging capacity for all extracts, with IC_50_ values ranging from 15.23 to 19.31 µg/mL. RN showed the strongest antioxidant activity, with the lowest IC_50_ value (15.23 ± 0.53 µg/mL), followed by CL (16.10 ± 1.03 µg/mL), ZM (17.00 ± 0.90 µg/mL), and ZO (19.31 ± 1.42 µg/mL). These results suggest that all four extracts possess potent antioxidant properties that may contribute to their therapeutic potential in topical application.

**Figure 1:**
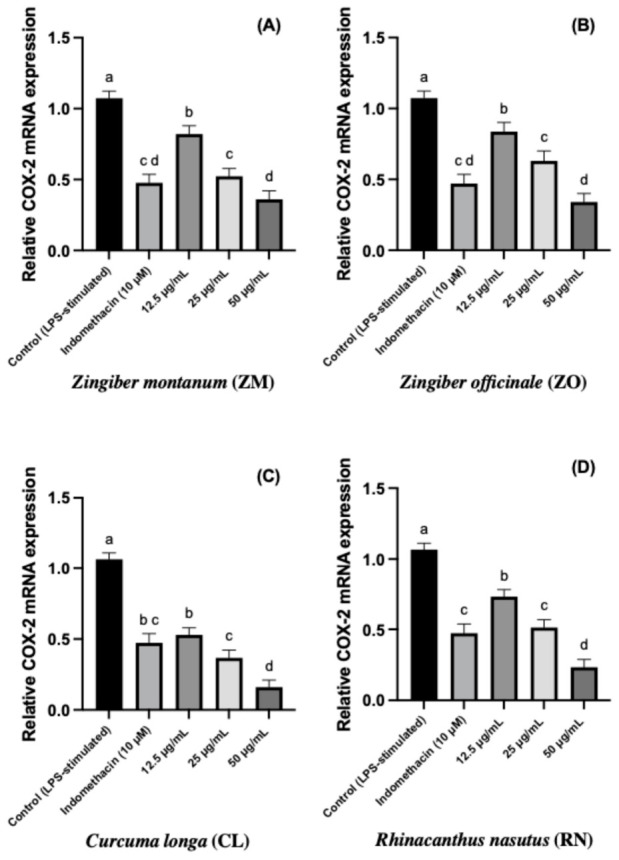
Concentration-dependent effects of four Thai medicinal plant extracts on relative COX-2 mRNA expression in LPS-stimulated RAW264.7 macrophages determined via quantitative real-time PCR (qRT-PCR). (**A**) *Zingiber montanum* (ZM), (**B**) *Zingiber officinale* (ZO), (**C**) *Curcuma longa* (CL), and (**D**) *Rhinacanthus nasutus* (RN). Cells were treated with each extract at concentrations of 12.5, 25, and 50 µg/mL. Untreated LPS-stimulated cells served as the negative control, whereas indomethacin served as the positive anti-inflammatory control. Relative COX-2 mRNA expression was normalized to the internal reference gene and expressed relative to the untreated control. Data are presented as mean ± standard deviation (SD) from three independent experiments (*n* = 3). Statistical significance was analyzed using one-way analysis of variance (ANOVA) followed by Tukey’s multiple comparison test. Different lowercase letters (a–d) above the bars indicate statistically significant differences among treatment groups within each panel (*p* < 0.05), whereas groups sharing at least one common letter are not significantly different (*p* ≥ 0.05).

Anti-inflammatory activity was evaluated by measuring relative COX-2 mRNA expression in LPS-stimulated RAW264.7 macrophages (Figure 1). All extracts significantly reduced COX-2 expression compared with the LPS-treated control (*p* < 0.05), with greater inhibition generally observed at higher concentrations (25–50 µg/mL). CL exhibited the strongest anti-inflammatory activity among the extracts, reducing COX-2 expression to approximately 0.15-fold of the control level at 50 µg/mL, comparable to or lower than that of the positive control, indomethacin. RN also showed pronounced inhibitory activity, reducing COX-2 expression to approximately 0.20-fold at 50 µg/mL. ZM and ZO showed concentration-dependent suppression of COX-2 expression, with the greatest inhibition observed at 50 µg/mL. Taken together, these results indicate that the selected Thai medicinal plants possess substantial antioxidant and anti-inflammatory activities and may serve as promising functional ingredients for veterinary topical formulations.

### 3.3. Antimicrobial Activity

Antibacterial activities of the selected Thai medicinal plant extracts were evaluated against bacterial pathogens commonly associated with dermatological infections in companion animals, including *Staphylococcus aureus* ATCC 25923, *Staphylococcus epidermidis* ATCC 12228, *Escherichia coli* ATCC 25922, and *Pseudomonas aeruginosa* ATCC 27853. Antimicrobial susceptibility was determined using the broth microdilution method following the recommendations of the Clinical and Laboratory Standards Institute (CLSI M07), and MIC and MBC values were interpreted according to the current CLSI Performance Standards for Antimicrobial Susceptibility Testing [61]. The results are summarized in Table 4.

Among the tested extracts, *Rhinacanthus nasutus* (RN) and *Zingiber montanum* (ZM) exhibited the strongest antibacterial activities. Both extracts inhibited *S. aureus* with MIC and MBC values of 8 μg/mL. Against *S. epidermidis*, both extracts demonstrated MIC values of 8 μg/mL and MBC values of 16 μg/mL. RN showed moderate activity against Gram-negative bacteria, exhibiting MIC values of 64 μg/mL against both *E. coli* and *P. aeruginosa*, whereas ZM demonstrated comparatively stronger activity against Gram-negative bacteria, with MIC values of 16 μg/mL against *E. coli* and 32 μg/mL against *P. aeruginosa*. In contrast, *Curcuma longa* (CL) exhibited limited antibacterial activity, demonstrating inhibitory effects only against MRSA and *S. epidermidis* (MIC = 64 μg/mL) but no detectable activity against *S. aureus*, *E. coli*, or *P. aeruginosa* within the concentration range tested.

To verify assay performance, standard antimicrobial agents were included as positive controls. Vancomycin demonstrated MIC values ranging from 0.5 to 1 μg/mL against *S. aureus* ATCC 25923 and *S. epidermidis* ATCC 12228, whereas gentamicin exhibited MIC values of 0.5 μg/mL against *S. epidermidis* ATCC 12228 and *P. aeruginosa* ATCC 27853. These MIC values were verified against the original laboratory records and were interpreted according to the CLSI M100 Performance Standards. Minor variations in MIC values may occur depending on bacterial strain characteristics, inoculum density, growth conditions, and experimental methodology. Therefore, the observed MIC values are considered acceptable for the reference strains employed in this study. Overall, RN and ZM showed the most promising antibacterial activities among the tested extracts and were therefore selected as the principal antibacterial ingredients for subsequent shampoo formulation.

Antifungal activities were evaluated against *Malassezia pachydermatis* ATCC 14522, *Microsporum canis* ATCC 36299, and *Microsporum gypseum* ATCC 14683, three fungal pathogens commonly associated with dermatological diseases in companion animals. MIC values are summarized in Table 4.

Among all tested extracts, *Zingiber officinale* (ZO) exhibited the strongest antifungal activity. Against *M. pachydermatis*, ZO demonstrated MIC and MFC values of 2 μg/mL, whereas MIC and MFC values against *M. gypseum* were 2 μg/mL and 16 μg/mL, respectively. Similarly, ZO showed potent activity against *M. canis*, exhibiting an MIC value of 8 μg/mL and an MFC value of 16 μg/mL, indicating broad-spectrum antifungal activity against both yeast and dermatophyte pathogens. Moderate antifungal activity was observed for *Curcuma longa* (CL) and *Zingiber montanum* (ZM), with MIC values ranging from 16 to 32 μg/mL against the tested fungi, whereas *Rhinacanthus nasutus* (RN) demonstrated comparatively weaker antifungal activity, particularly against *M. gypseum*, for which both MIC and MFC values reached 64 μg/mL.

Clotrimazole served as the reference antifungal agent and exhibited MIC values of 4 μg/mL against *M. pachydermatis*, 2 μg/mL against *M. canis*, and 1 μg/mL against *M. gypseum*, confirming the validity and reliability of the antifungal susceptibility assay.

Collectively, these findings demonstrate that RN and ZM exhibited the strongest antibacterial activities, whereas ZO possessed the greatest antifungal activity. These complementary biological properties provided the scientific rationale for selecting these extracts for incorporation into the prototype shampoo formulation. The present study did not evaluate pharmacodynamic interactions among the combined extracts; therefore, no conclusions regarding synergistic or additive effects can be drawn.

### 3.4. Evaluation and Formulation of Shampoo

The developed dog and cat shampoo formulations exhibited desirable physicochemical characteristics and retained their biological activities after incorporation of the selected herbal extracts and RPO. Both formulations appeared as homogeneous gels, with no visible phase separation, precipitation, or creaming throughout the evaluation period. Following centrifugation, neither the dog nor cat shampoo formulation exhibited visible phase separation, precipitation, or color change. Furthermore, no substantial alterations in pH or viscosity were observed after centrifugation, indicating the satisfactory preliminary physical stability of both formulations under the experimental conditions (Table 5). These findings suggest that incorporation of the herbal extracts and red palm oil did not adversely affect the physical homogeneity of the formulations during short-term stress testing. However, centrifugation represents only an initial screening method for formulation stability and cannot substitute for comprehensive long-term stability evaluation.

The dog shampoo appeared as an orange-red gel, whereas the cat shampoo had a lighter orange appearance. The pH values of dog and cat shampoos were 7.75 ± 0.03 and 7.07 ± 0.04, respectively, within the acceptable range for companion animal skin care. Viscosity values were 2850 ± 55 cP for the dog shampoo and 2620 ± 60 cP for the cat shampoo, indicating suitable rheological properties for topical application.

Antibacterial activity of both formulations was retained after formulation (Table 4). MIC values against *S. aureus* and *S. epidermitis* were 8 µg/mL for both shampoos. Against Gram-negative bacteria, MIC values of 16 µg/mL and 32 µg/mL were observed against EC and PA, respectively, with no significant difference between the two formulations, indicating that incorporation into the shampoo base did not adversely affect antibacterial efficacy.

Both formulations also retained antifungal activity against dermatological fungal pathogens. MIC values against *M. canis*, *M. gypseum*, and *M. pachydermatis* were 32, 64, and 16 µg/mL, respectively. Antifungal activity of the formulated products was comparable to that of the corresponding crude extracts, suggesting good compatibility between the active ingredients and the shampoo matrix.

Antibacterial inhibition percentages of dog and cat shampoo formulations were 92.4 ± 1.3% and 90.8 ± 1.5%, respectively, while antifungal inhibition percentages were 88.6 ± 1.9% and 87.3 ± 2.1%, respectively. Overall, both formulations showed favorable physicochemical stability and retained substantial antimicrobial and antifungal activity, supporting their potential as topical products for companion animal skin care.

## 4. Discussion

This study provides a proof of concept for shampoo formulations that integrate red palm oil (RPO) recovered from oil palm mesocarp residue with extracts of *Rhinacanthus nasutus* (RN), *Curcuma longa* (CL), *Zingiber montanum* (ZM), and *Zingiber officinale* (ZO) for companion animal skin care. The extracts showed antioxidant, anti-inflammatory, antibacterial, and antifungal activities, and the final dog and cat shampoo prototypes retained measurable antimicrobial activity after incorporation into the surfactant base. The principal novelty of this work is the combined use of residue-derived RPO and selected Thai medicinal plants in species-adjusted shampoo prototypes. Nevertheless, the findings represent an in vitro formulation study and should not yet be interpreted as evidence of clinical efficacy or dermal safety in dogs and cats.

The relatively high extraction yields of RPO (42.02%) and ZM (34.00%) indicate that microwave-assisted extraction (MAE) was suitable for recovering extractable constituents under the conditions used. However, extraction yield was not directly associated with biological activity, because the activity of a botanical extract depends on both the abundance and chemical characteristics of its constituents [62,63]. CL had the highest curcumin concentration and total phenolic content, whereas RN produced the lowest DPPH IC_50_ value. This difference suggests that antioxidant performance was influenced by qualitative phytochemical composition rather than the total phenolic concentration alone. Because extract combinations were not evaluated separately in the antioxidant assay, the present results do not establish synergistic interactions among the phytochemicals.

RPO contained substantial β-carotene and vitamin E concentrations and was dominated by palmitic and oleic acids. These constituents may support topical antioxidant and emollient functions by limiting lipid oxidation and contributing to the lipid phase of the formulation [25,28]. However, the statement that MAE preserved fatty-acid integrity should be interpreted cautiously because this study did not directly compare the same raw material before and after extraction under alternative processing conditions. The use of palm fruit residue represents a potentially valuable by-product valorization strategy, but environmental sustainability was not quantified in this study. Future assessments should report the mass of residue diverted per unit of RPO produced, solvent and energy consumption, extraction waste, carbon footprint, and production cost to determine whether the process provides a measurable environmental and economic benefit.

All four herbal extracts reduced COX-2 mRNA expression, with stronger suppression generally observed at higher concentrations, and particularly pronounced activity was observed for CL. This result is consistent with reports that curcumin- and ginger-derived compounds can modulate inflammatory signaling involving reactive oxygen species, NF-κB, and COX-2 [64,65,66]. Nevertheless, reduced COX-2 transcript abundance alone does not confirm inhibition of the complete inflammatory pathway. Measurements of the COX-2 protein, prostaglandin E_2_ production, additional inflammatory mediators, and cell viability would be needed to distinguish a specific anti-inflammatory effect from nonspecific cellular toxicity.

Assuming that the intended MIC unit is micrograms per milliliter, the antibacterial findings can be compared with previous reports. The RN MIC of 8 µg/mL against *S. aureus* and *S. epidermidis* is consistent with the 8–16 µg/mL range reported for a standardized rhinacanthin-rich RN extract [67]. The ZM MIC of 8 µg/mL against *S. aureus* and MRSA was lower than the 64–256 µg/mL values previously reported for nonpolar ZM crude extracts and the 32–128 µg/mL range reported for isolated terpenes [68]. Such differences may reflect plant origin, extract composition, solvent, marker compound concentration, strain susceptibility, and assay conditions.

ZO also exhibited the strongest antifungal activity, with an MIC of 2 µg/mL against *M. pachydermatis* and *M. gypseum*. Published values for ginger preparations vary widely. For example, *Z. officinale* essential oils from different geographical sources showed an MIC of 313 µg/mL against *M. gypseum* [69], whereas another study reported an MIC of 0.06 µL/mL for ginger essential oil against the same dermatophyte [70]. These values are not directly interchangeable because the studies used different extract types, concentration units, fungal isolates, and susceptibility protocols. The low MIC obtained in the present study is therefore promising but should be confirmed using independently prepared extract batches, multiple clinical isolates, and standardized organism-specific methods. In particular, *M. pachydermatis* is a yeast, whereas *M. canis* and *M. gypseum* are dermatophytes, and their culture and susceptibility requirements should be reported separately.

The final formulations were homogeneous and had pH and viscosity values suitable for preliminary topical product development. The present study employed centrifugation as a preliminary stress test to evaluate the physical stability of the shampoo formulations. Although neither formulation exhibited visible phase separation nor significant changes in physicochemical characteristics following centrifugation, these observations should not be interpreted as evidence of long-term product stability. Comprehensive stability evaluation, including accelerated stability testing under ICH Q1A(R2) conditions, real-time storage studies, preservative challenge testing [71], oxidative stability of carotenoids and tocopherols, and packaging compatibility studies, will be conducted during subsequent product development. Incorporation into the shampoo base retained antimicrobial activity, but the data do not support the statement that potency remained unchanged for every organism. Relative to the most active individual extract, the formulation’s MIC increased from 2 to 8 µg/mL against *S. aureus* and *S. epidermidis*, from 2 to 16 µg/mL against *M. pachydermatis*, and from 2 to 64 µg/mL against *M. gypseum*. These shifts may reflect the dilution of the active extract, interactions with surfactants or other ingredients, or differences in the amount of active marker compounds delivered during testing. A shampoo-base-only control, active-equivalent concentration calculations, contact-time assays, and time-kill studies are needed to determine how the formulation matrix affects activity. Moreover, measurements obtained at a single or incompletely described storage interval demonstrate initial physicochemical compatibility rather than long-term stability.

The antimicrobial activity of a multi-extract shampoo does not by itself demonstrate synergism. Because the individual extracts and their combinations were not examined using checkerboard assays, fractional inhibitory concentration indices, or time-kill analysis, the observed activity could be additive, indifferent, antagonistic, or largely attributable to the most active component. Claims of synergistic activity, reduced resistance development, or suitability for long-term use should therefore be avoided until they are experimentally demonstrated.

Although several ingredients in the formulation have demonstrated topical biological activity and acceptable tolerability in experimental models, species-specific evidence regarding their dermal safety and incidental ingestion, particularly in cats, remains limited. This issue is especially relevant because cats may ingest shampoo residues remaining on the skin or coat during post-bathing grooming [72]. Cats also have species-specific limitations in the glucuronidation of several phenolic xenobiotics, which may increase their susceptibility to certain plant-derived compounds. However, this metabolic characteristic alone does not indicate that the botanical ingredients used in the present formulation are toxic to cats [73].

Available non-feline evidence supports the potential topical application of several individual ingredients. Topical curcumin and ginger extracts improved wound healing without visible skin irritation in hairless rats [74], while a topical preparation of cassumunar ginger exhibited anti-inflammatory activity and enhanced skin permeation in experimental models [75]. In dogs with localized demodicosis, topical treatment combining Dipterocarpus alatus oil with Rhinacanthus nasutus leaf extracts improved dermatological lesions, with no allergic or clinical signs observed during the treatment period [76]. Similarly, a tocotrienol-rich nanoemulsion derived from red palm oil promoted wound healing in preclinical models [77]. Nevertheless, these findings cannot confirm the safety of the complete formulation in cats or dogs because the available studies involved different species, preparations, concentrations, and exposure conditions. Therefore, species-specific in vivo studies evaluating dermal and ocular irritation, repeated topical exposure, systemic absorption, accidental ingestion, rinsability, and therapeutic efficacy are required before veterinary application.

Several limitations should be considered. This study was restricted to in vitro assays and did not evaluate cytotoxicity, skin irritation, sensitization, or barrier effects in canine- or feline-derived keratinocytes, fibroblasts, reconstructed skin, or ex vivo skin. Long-term and accelerated stability, oxidation of RPO carotenoids and tocopherols, preservative efficacy, repeated-batch chemical standardization, packaging compatibility, and cost-effectiveness were also not assessed. Future research should include checkerboard and time-kill studies of extract combinations; cytotoxicity and irritation testing in pet-derived cells and ex vivo skin; standardized marker compound and batch-variation analyses; real-time and accelerated stability studies; and controlled in vivo studies in dogs and cats with bacterial pyoderma, Malassezia-associated dermatitis, or dermatophytosis. Clinical trials should assess lesion severity, microbial burden, recurrence, tolerability, owner-reported outcomes, and the effect of repeated use on the normal skin microbiota.

One important limitation of the present study is that the biological activities of the combined herbal formulation were evaluated without assessing pharmacodynamic interactions among the individual extracts. Although the formulation demonstrated promising antibacterial, antifungal, antioxidant, and anti-inflammatory properties, these findings should not be interpreted as evidence of synergistic interactions. Confirmation of synergistic, additive, or antagonistic effects requires dedicated combination studies, including checkerboard assays with fractional inhibitory concentration index (FICI) determination and time-kill kinetics. These investigations are planned as part of future product optimization and mechanistic evaluation.

Another limitation of the present study is that the product’s shelf life could not be estimated because long-term and accelerated stability studies were not performed. Future studies will include accelerated and real-time stability testing to establish the product’s shelf life and storage stability. Moreover, further in vivo studies are required to evaluate the safety of this treatment before clinical application.

## 5. Conclusions

This study provides a proof of concept for shampoo containing RPO and Thai herbs for treating skin infections in pets. RN, ZM, and ZO showed antimicrobial activity, while CL showed prominent antioxidant and COX-2-suppressive effects. The formulated shampoos had an acceptable initial appearance, pH, and viscosity and retained measurable antibacterial and antifungal activities after the active ingredients were incorporated. This suggests that the RPO and multiple Thai medicinal-plant shampoos can be used for alternative treatment of skin infections. However, standardized combination testing, safety evaluation in canine and feline skin models, stability and batch-consistency studies, and controlled clinical trials are required before the formulations can be recommended for routine veterinary use.

## Figures and Tables

**Table 1:** Quantification of major phytochemical marker compounds in Thai medicinal plant extracts.

Extract	Marker Compound	Concentration (mg/mL)
*Rhinacanthus nasutus* (RN)	Rhinacanthin	1.78 ± 0.26
*Curcuma longa* (CL)	Curcumin	5.34 ± 0.71
*Zingiber montanum* (ZM)	Terpinen-4-ol	2.60 ± 1.67
*Zingiber officinale* (ZO)	6-Gingerol	1.30 ± 1.85

Values are expressed as mean ± SD (*n* = 3).

**Table 2:** Fatty acid composition of mesocarp-derived red palm oil (RPO) compared with commercial crude red palm oil.

Fatty Acid	Mesocarp-Derived RPO (% Total Fatty Acids)	Commercial Crude RPO (% Total Fatty Acids)
Palmitic acid (C16:0)	54.36 ± 0.82 ^a^	40.17 ± 0.64 ^b^
Stearic acid (C18:0)	4.08 ± 0.11 ^a^	4.85 ± 0.13 ^b^
Oleic acid (C18:1)	29.74 ± 0.67 ^b^	40.08 ± 0.81 ^a^
Linoleic acid (C18:2)	6.60 ± 0.19 ^b^	10.27 ± 0.25 ^a^
Linolenic acid (C18:3)	0.17 ± 0.01 ^b^	0.35 ± 0.02 ^a^
Arachidic acid (C20:0)	0.33 ± 0.02 ^a^	0.38 ± 0.03 ^a^
Eicosenoic acid (C20:1)	0.10 ± 0.01 ^b^	0.14 ± 0.01 ^a^
Palmitic acid (C16:0)	54.36 ± 0.82 ^a^	40.17 ± 0.64 ^b^
Stearic acid (C18:0)	4.08 ± 0.11 ^a^	4.85 ± 0.13 ^b^
Oleic acid (C18:1)	29.74 ± 0.67 ^b^	40.08 ± 0.81 ^a^

Values are expressed as mean ± SD (*n* = 3). Different superscript letters within the same row indicate significant differences between mesocarp-derived RPO and commercial crude RPO (*p* < 0.05).

**Table 3:** Total phenolic content and antioxidant activity of Thai medicinal plant extracts.

Extract	Total Phenolic Content (mg GAE/g DW)	DPPH IC_50_ (µg/mL)
*Rhinacanthus nasutus* (RN)	121.94 ± 7.69 ^b^	15.23 ± 0.53 ^c^
*Curcuma longa* (CL)	242.00 ± 5.12 ^a^	16.10 ± 1.03 ^bc^
*Zingiber montanum* (ZM)	117.34 ± 4.23 ^b^	17.00 ± 0.90 ^b^
*Zingiber officinale* (ZO)	89.45 ± 4.53 ^c^	19.31 ± 1.42 ^a^

Values are expressed as mean ± SD (*n* = 3). Different superscript letters within the same column indicate significant differences (*p* < 0.05).

**Table 4:** Physicochemical properties and biological activities of dog and cat shampoo formulations.

Parameter	Dog Shampoo	Cat Shampoo
Appearance	Homogeneous orange-red gel	Homogeneous light-orange gel
Phase separation	Not observed	Not observed
pH	7.75 ± 0.03	7.07 ± 0.04
Viscosity (cP)	2850 ± 55	2620 ± 60
Antibacterial inhibition (%)	92.4 ± 1.3	90.8 ± 1.5
Antifungal inhibition (%)	88.6 ± 1.9	87.3 ± 2.1
MIC against *S. aureus* (µg/mL)	8	8
MIC against *S. epidermitis* (µg/mL)	8	8
MIC against *E. coli* (µg/mL)	16	16
MIC against *P. aeruginosa* (µg/mL)	32	32
MIC against *M. pachydermatis* (µg/mL)	16	16
MIC against *M. canis* (µg/mL)	32	32
MIC against *M. gypseum* (µg/mL)	64	64

Values are expressed as mean ± SD (*n* = 3).

**Table 5:** Preliminary physicochemical stability assessment of prototype dog and cat shampoo formulations before and after centrifugation.

Parameter	Dog Shampoo (Before)	Dog Shampoo (After Centrifugation)	Cat Shampoo (Before)	Cat Shampoo (After Centrifugation)
Appearance	Homogeneous orange-red gel	No visible change	Homogeneous light-orange gel	No visible change
Phase separation	None	None	None	None
pH	7.75 ± 0.03	7.73 ± 0.04	7.07 ± 0.04	7.05 ± 0.05
Viscosity (cP)	2850 ± 55	2838 ± 61	2620 ± 60	2608 ± 58

Values are expressed as mean ± SD (*n* = 3). No visible phase separation was observed following centrifugation. Physicochemical measurements were performed immediately before and after centrifugation.

## Data Availability

The data presented in this study are available on request from the corresponding author, due to privacy and personal constraints of the research team.

## Abbreviations

The following abbreviations are used in this manuscript:

RPO	Red palm oil
RN	Rhinacanthus nasutus
CL	Curcuma longa
ZM	Zingiber montanum
ZO	Zingiber officinale
MAE	Microwave-assisted extraction
TPC	Total phenolic content
DPPH	2,2-diphenyl-1-picrylhydrazyl
HPLC	High-performance liquid chromatography
GC–MS	Gas chromatography–mass spectrometry
FAMEs	Fatty acid methyl esters
AMR	Antimicrobial resistance
COX-2	Cyclooxygenase-2
MIC	Minimum inhibitory concentration
MBC	Minimum bactericidal concentration
MFC	Minimum fungicidal concentration
CFU	Colony-forming unit
LOD	Limit of detection
LOQ	Limit of quantification
SD	Standard deviation

## Appendix A

**Table A1:** Composition of dog and cat shampoo formulations.

Ingredient	Dog Shampoo (% *w*/*w*)	Cat Shampoo (% *w*/*w*)
Red palm oil (RPO)	5.0	5.0
RN extract	1.0	1.0
CL extract	0.5	0.5
ZM extract	1.0	1.0
ZO extract	1.0	1.0
Sodium lauryl ether sulfate (SLES)	15.0	12.0
Cocamidopropyl betaine	8.0	10.0
Glycerin	3.0	3.0
Potassium sorbate	0.20	0.20
Citric acid	q.s. to pH 7.75	q.s. to pH 7.07
Purified water	q.s. to 100	q.s. to 100

**Table A2:** Stability testing conditions for the shampoo formulation.

Test Parameter	Condition
Real-time storage	25 ± 2 °C
Accelerated storage	40 ± 2 °C
Storage duration	3 months
Centrifugation test	3000 rpm, 30 min
Parameters evaluated	Appearance, color, odor, pH, viscosity, phase separation
Replicates	*n* = 3

## References

[1] Knight A. (2023). The relative benefits for environmental sustainability of vegan diets for dogs, cats and people. PLoS ONE.

[2] Ellis A., Stanton S.C., Hawkins R.D., Loughnan S. (2024). The link between the nature of the human–companion animal relationship and well-being outcomes in companion animal owners. Animals.

[3] De Silva L. (2025). Cat vs. Dog People: Perceived Well-Being of Companion Animals as a Function of Species Preferences and Documented Interest. Ph.D. Thesis.

[4] Tu A.Y., Springer C.M., Albright J.D. (2024). Evaluation of Characteristics Associated with Self-Identified Cat or Dog Preference in Pet Owners and Correlation of Preference with Pet Interactions and Care: An Exploratory Study. Animals.

[5] Rand J., Ahmadabadi Z., Norris J., Franklin M. (2023). Attitudes and beliefs of a sample of Australian dog and cat owners towards pet confinement. Animals.

[6] Alabbody H. (2024). The Control and Preventative Measures for the Health Problems of Pets. J. Anim. Health Prod..

[7] Miller J., Simpson A., Bloom P., Diesel A., Friedeck A., Paterson T., Wisecup M., Yu C.-M. (2023). 2023 AAHA management of allergic skin diseases in dogs and cats guidelines. J. Am. Anim. Hosp. Assoc..

[8] Araújo D., Silva A.R., Fernandes R., Serra P., Barros M.M., Campos A.M., Oliveira R., Silva S., Almeida C., Castro J. (2024). Emerging approaches for mitigating biofilm-formation-associated infections in farm, wild, and companion animals. Pathogens.

[9] Yılmaz N.K., Baş B. (2024). Superficial pyoderma in cats and dogs: A retrospective clinical study. Ank. Üniversitesi Vet. Fakültesi Derg..

[10] Alautaish H.H., Naji H.A., Saud Z.A.H. (2024). Clinical study of common bacterial, fungal and parasitic skin diseases in cats. Adv. Life Sci..

[11] Hobi S., Cafarchia C., Romano V., Barrs V.R. (2022). Malassezia: Zoonotic implications, parallels and differences in colonization and disease in humans and animals. J. Fungi.

[12] Kundu R., Bansal Y., Singla N. (2024). The zoonotic potential of fungal pathogens: Another dimension of the one health approach. Diagnostics.

[13] Yurayart C., Niae S., Limsivilai O., Thengchaisri N., Sattasathuchana P. (2024). Comparative analysis of the distribution and antifungal susceptibility of yeast species in cat facial hair and human nails. Sci. Rep..

[14] Sattasathuchana P., Bumrungpun C., Thengchaisri N. (2020). Comparison of subclinical dermatophyte infection in short-and long-haired cats. Vet. World.

[15] Sykes J.E., Damborg P. (2024). Antimicrobial Therapy in Dogs and Cats. Antimicrobial Therapy in Veterinary Medicine.

[16] Malinovská Z., Fekeová L. (2023). Dermatological diseases in dogs: A survey in veterinary facilities. Folia Vet..

[17] Robbins S.N., Goggs R., Kraus-Malett S., Goodman L. (2024). Effect of institutional antimicrobial stewardship guidelines on prescription of critically important antimicrobials for dogs and cats. J. Vet. Intern. Med..

[18] Niae S., Yurayart C., Thengchaisri N., Sattasathuchana P. (2021). Prevalence and in vitro antifungal susceptibility of commensal yeasts in the external ear canal of cats. BMC Vet. Res..

[19] Ortiz-Millán G. (2025). One Health in a globalized world: Challenges and responses to zoonotic threats. Glob. Bioeth..

[20] Khan M.F. (2025). Novel Approaches in Treating Skin Infections in Cats and Dogs: Exploring Antibiotic Resistance and Alternative Therapies. ABR.

[21] Marco-Fuertes A., Marin C., Lorenzo-Rebenaque L., Vega S., Montoro-Dasi L. (2022). Antimicrobial resistance in companion animals: A new challenge for the One Health approach in the European Union. Vet. Sci..

[22] Stefanetti V., Passamonti F., Rampacci E. (2024). Antimicrobial strategies proposed for the treatment of *S. pseudintermedius* and other dermato-pathogenic Staphylococcus spp. in companion animals: A narrative review. Vet. Sci..

[23] Tadee P., Chansakaow S., Tipduangta P., Tadee P., Khaodang P., Chukiatsiri K. (2023). Essential oil pharmaceuticals for killing ectoparasites on dogs. J. Vet. Sci..

[24] Bava R., Bulotta R.M., Castagna F., Ruga S., Lupia C., Conforti F., Statti G., Crupi R., Musella V., Palma E. (2026). Essential Oils for Flea and Tick Control in Companion Animals: A Critical Review of Efficacy, Safety, Resistance Mitigation and Integrated Pest Management. Antibiotics.

[25] Madoromae H., Lertcanawanichakul M. (2025). Red Palm Oil: Nutritional Composition, Bioactive Properties, and Potential Applications in Health and Cosmetics: A Narrative Review. Molecules.

[26] Lertcanawanichakul M., Sahabuddeen T., Madoromae H., Jankhlay S. (2025). Red Palm Oil as a Bioactive Ingredient for Cosmeceutical Applications: Antioxidant, Antimicrobial, and Skin-Protective Properties. Preprints.

[27] Papadopoulou S.N.A., Adamantidi T., Kranas D., Cholidis P., Anastasiadou C., Tsoupras A. (2025). A comprehensive review on the valorization of bioactives from marine animal by-products for health-promoting, biofunctional cosmetics. Mar. Drugs.

[28] Stanescu C., Chiscop I., Mihalache D., Popa F., Tamas C., Stoleriu G. (2025). Skin aging and carotenoids: A systematic review of their multifaceted protective mechanisms. Nutrients.

[29] John Martin J.J., Wang Q., Hou M., Li X., Liu X., Fang Z., Sun C., Cao H. (2026). Palm oil: A review on nutritional composition, processing, contaminants, and sustainability frameworks in the food system. Front. Plant Sci..

[30] Brimson J.M., Prasanth M.I., Malar D.S., Brimson S., Tencomnao T. (2020). *Rhinacanthus nasutus* “tea” infusions and the medicinal benefits of the constituent phytochemicals. Nutrients.

[31] Khatun M., Nur M.A., Biswas S., Khan M., Amin M.Z. (2021). Assessment of the anti-oxidant, anti-inflammatory and anti-bacterial activities of different types of turmeric (*Curcuma longa*) powder in Bangladesh. J. Agric. Food Res..

[32] Devkota H.P., Paudel K.R., Hassan M.M., Dirar A.I., Das N., Adhikari-Devkota A., Echeverría J., Logesh R., Jha N.K., Singh S.K. (2021). Bioactive compounds from Zingiber montanum and their pharmacological activities with focus on zerumbone. Appl. Sci..

[33] Ayustaningwarno F., Anjani G., Ayu A.M., Fogliano V. (2024). A critical review of Ginger’s (*Zingiber officinale*) antioxidant, anti-inflammatory, and immunomoFront Nutrdulatory activities. Front. Nutr..

[34] Alem W.T. (2024). Effect of herbal extracts in animal nutrition as feed additives. Heliyon.

[35] Chandra S., Saklani S., Kumar P., Kim B., Coutinho H.D. (2022). Nutraceuticals: Pharmacologically active potent dietary supplements. Biomed. Res. Int..

[36] Saeed M., Munawar M., Bi J.B., Ahmed S., Ahmad M.Z., Kamboh A.A., Arain M.A., Naveed M., Chen H. (2024). Promising phytopharmacology, nutritional potential, health benefits, and traditional usage of *Tribulus terrestris* L. herb. Heliyon.

[37] Wang E., Wu P., Xu J., Hong X., Zhao N., Sun T., Zhang T., Xu Z., Yang C. (2026). Effects of Traditional Chinese Herbal Extracts on Tear Staining, Iron Status, Immune Function, and Antioxidant Capacity in Dogs. Animals.

[38] Ayele G., Admassu H., Mosisa G., Desalegn A., Abeje M. (2025). Emerging techniques for catechin extraction from green tea (*Camellia sinensis*): Extraction technologies, functional potential, Toxicology, and food-industry applications: A systematic review. Cogent Food Agric..

[39] Singh J., Rasane P., Kaur R., Kaur H., Garg R., Kaur S., Ercisli S., Choudhary R., Skrovankova S., Mlcek J. (2023). Valorization of grape (*Vitis vinifera*) leaves for bioactive compounds: Novel green extraction technologies and food-pharma applications. Front. Chem..

[40] Nonglait D.L., Gokhale J.S. (2024). Review insights on the demand for natural pigments and their recovery by emerging microwave-assisted extraction (MAE). Food Bioprocess Technol..

[41] López-Salazar H., Camacho-Díaz B.H., Ocampo M.L.A., Jiménez-Aparicio A.R. (2023). Microwave-assisted extraction of functional compounds from plants: A Review. BioResources.

[42] Andry M., Ligo A., Anggi R.D., Pradita D., Luthvia L., Nasution M.A., Pertiwi N.N., Arifin A. (2025). The Effect of Different Methods of Maceration and Microwave Assisted Extraction (MAE) on Determining Flavonoid Contents of Total Figs (*Ficus racemosa* L). J. Pharm. Sci..

[43] Imane G., Hemmami H., Ben Amor I., Zeghoud S., Ben Seghir B., Hammoudi R. (2023). Different methods of extraction of bioactive compounds and their effect on biological activity: A review. Int. J. Second. Metab..

[44] Chemat F., Cravotto G. (2013). Microwave-Assisted Extraction for Bioactive Compounds.

[45] Dhotre I. (2025). A comprehensive review on progression and innovations in microwave assisted extraction technology for essential oils. J. Chem. Technol. Biotechnol..

[46] Ferrara D., Beccaria M., Cordero C.E., Purcaro G. (2023). Microwave-assisted extraction in closed vessel in food analysis. J. Sep. Sci..

[47] Nithya S., Krishnan R.R., Rao N.R., Naik K., Praveen N., Vasantha V.L., Cruz J.N. (2023). Microwave-Assisted Extraction of Phytochemicals. Drug Discovery and Design Using Natural Products.

[48] Yuan Y., Zhang J., Fan J., Clark J., Shen P., Li Y., Zhang C. (2018). Microwave assisted extraction of phenolic compounds from four economic brown macroalgae species and evaluation of their antioxidant activities and inhibitory effects on α-amylase, α-glucosidase, pancreatic lipase and tyrosinase. Food Res. Int..

[49] Subramanyam G.B., Parrish D.B. (1976). Colorimetric reagents for determining vitamin A in feeds and foods. J. Assoc. Off. Anal. Chem..

[50] Christie W.W. (1989). Gas Chromatography and Lipids.

[51] American Oil Chemists’ Society (2017). Official Method Ce 2–66: Preparation of Methyl Esters of Fatty Acids. Official Methods and Recommended Practices of the AOCS.

[52] Association of Official Analytical Collaboration International (1995). Official Method 963.22: Methyl Esters of Fatty Acids in Oils and Fats—Gas Chromatographic Method. Official Methods of Analysis of AOAC International.

[53] Brand-Williams W., Cuvelier M.E., Berset C. (1995). Use of a free radical method to evaluate antioxidant activity. LWT-Food Sci. Technol..

[54] Sirivibulkovit K., Nouanthavong S., Sameenoi Y. (2018). Paper-based DPPH Assay for Antioxidant Activity Analysis. Anal. Sci..

[55] CLSI (2020). Methods for Dilution Antimicrobial Susceptibility Tests for Bacteria That Grow Aerobically. CLSI Guideline M07-A10.

[56] CLSI (2017). M38-A2: Reference Method for Broth Dilution Antifungal Susceptibility Testing of Filamentous Fungi.

[57] Hnilica K.A., Patterson A.P., Patterson A.P., Hnilica K.A. (2017). Chapter 1-Differential Diagnoses. Small Animal Dermatology.

[58] Draelos Z.D. (2018). The science behind skin care: Moisturizers. J. Cosmet. Dermatol..

[59] Proksch E., Brandner J.M., Jensen J.M. (2008). The skin: An indispensable barrier. Exp. Dermatol..

[60] Sarker S.D., Nahar L., Kumarasamy Y. (2007). Microtitre plate-based antibacterial assay incorporating resazurin as an indicator of cell growth, and its application in the in vitro antibacterial screening of phytochemicals. Methods.

[61] CLSI (2026). M100: Performance Standards for Antimicrobial Susceptibility Testing.

[62] Platzer M., Kiese S., Tybussek T., Herfellner T., Schneider F., Schweiggert-Weisz U., Eisner P. (2022). Radical scavenging mechanisms of phenolic compounds: A quantitative structure-property relationship (QSPR) study. Front. Nutr..

[63] Gil-Martín E., Forbes-Hernández T., Romero A., Cianciosi D., Giampieri F., Battino M. (2022). Influence of the extraction method on the recovery of bioactive phenolic compounds from food industry by-products. Food Chem..

[64] Leclercq I.A., Farrell G.C., Sempoux C., dela Peña A., Horsmans Y. (2004). Curcumin inhibits NF-κB activation and reduces the severity of experimental steatohepatitis in mice. J. Hepatol..

[65] Li X.-H., McGrath K.C., Tran V.H., Li Y.-M., Duke C.C., Roufogalis B.D., Heather A.K. (2013). Attenuation of proinflammatory responses by S-[6]-Gingerol via inhibition of ROS/NF-Kappa B/COX2 activation in HuH7 cells. Evid. Based Complement Altern. Med..

[66] Yuandani, Jantan I., Haque M.A., Rohani A.S., Nugraha S.E., Salim E., Septama A.W., Juwita N.A., Khairunnisa N.A., Nasution H.R. (2023). Immunomodulatory effects and mechanisms of the extracts and secondary compounds of Zingiber and Alpinia species: A review. Front. Pharmacol..

[67] Puttarak P., Charoonratana T., Panichayupakaranant P. (2010). Antimicrobial activity and stability of rhinacanthins-rich Rhinacanthus nasutus extract. Phytomedicine.

[68] Siddique H., Pendry B., Rahman M.M. (2019). Terpenes from Zingiber montanum and their screening against multi-drug resistant and methicillin resistant Staphylococcus aureus. Molecules.

[69] Kumar Poudel D., Dangol S., Rokaya A., Maharjan S., Kumar Ojha P., Rana J., Dahal S., Timsina S., Dosoky N.S., Satyal P. (2022). Quality assessment of Zingiber officinale Roscoe essential oil from Nepal. Nat. Prod. Commun..

[70] Sharma M., Sharma R. (2011). Synergistic antifungal activity of *Curcuma longa* (turmeric) and *Zingiber officinale* (ginger) essential oils against dermatophyte infections. J. Essent. Oil-Bear. Plants.

[71] (2019). Cosmetics—Microbiology—Evaluation of the Antimicrobial Protection of a Cosmetic Product.

[72] Kim H.S., Hong J.S., Park C.W., Cho K.H., Kim Y.Y. (2019). Evaluation of grooming behaviour and apparent digestibility method in cats. J. Feline Med. Surg..

[73] Court M.H. (2013). Feline drug metabolism and disposition: Pharmacokinetic evidence for species differences and molecular mechanisms. Vet. Clin. N. Am. Small Anim. Pract..

[74] Bhagavathula N., Warner R.L., DaSilva M., McClintock S.D., Barron A., Aslam M.N., Johnson K.J., Varani J. (2009). A combination of curcumin and ginger extract improves abrasion wound healing in corticosteroid-impaired hairless rat skin. Wound Repair Regen..

[75] Priprem A., Janpim K., Nualkaew S., Mahakunakorn P. (2016). Topical Niosome Gel of *Zingiber cassumunar Roxb*. Extract for Anti-inflammatory Activity Enhanced Skin Permeation and Stability of Compound D. AAPS PharmSciTech.

[76] Artchayasawat A., Boueroy P., Boonmars T., Pumhirunroj B., Sriraj P., Aukkanimart R., Boonjaraspinyo S., Pitaksakulrat O., Ratanasuwan P., Suwannatrai A. (2021). Efficacy of *Dipterocarpus alatus* oil combination with *Rhinacanthus nasutus* leaf and *Garcinia mangostana* pericarps against canine demodicosis. Vet. World.

[77] Chong W.T., Tan C.P., Cheah Y.K., Lai O.M. (2022). In-vitro and in-vivo evaluations of tocotrienol-rich nanoemulsified system on skin wound healing. PLoS ONE.

